# Cellular Mechanisms Contributing to the Functional Heterogeneity of GABAergic Synapses

**DOI:** 10.3389/fnmol.2019.00187

**Published:** 2019-08-13

**Authors:** Benjamin F. N. Campbell, Shiva K. Tyagarajan

**Affiliations:** Institute of Pharmacology and Toxicology, University of Zurich, Zurich, Switzerland

**Keywords:** homeostatic plasticity, postsynaptic density, interneurons, gephyrin, post-transcriptional regulation, post-translational modifications

## Abstract

GABAergic inhibitory neurotransmission contributes to diverse aspects of brain development and adult plasticity, including the expression of complex cognitive processes. This is afforded for in part by the dynamic adaptations occurring at inhibitory synapses, which show great heterogeneity both in terms of upstream signaling and downstream effector mechanisms. Single-particle tracking and live imaging have revealed that complex receptor-scaffold interactions critically determine adaptations at GABAergic synapses. Super-resolution imaging studies have shown that protein interactions at synaptic sites contribute to nano-scale scaffold re-arrangements through post-translational modifications (PTMs), facilitating receptor and scaffold recruitment to synaptic sites. Additionally, plasticity mechanisms may be affected by the protein composition at individual synapses and the type of pre-synaptic input. This mini-review article examines recent discoveries of plasticity mechanisms that are operational within GABAergic synapses and discusses their contribution towards functional heterogeneity in inhibitory neurotransmission.

## Introduction

The plasticity of individual synapses occurs downstream of activity or neuro-modulatory signaling and must be reconciled with homeostatic mechanisms to maintain overall network function (Abbott and Nelson, 2000). The inherent variability in functional connectivity between different neuronal cell types within or between brain regions is becoming apparent. However, even at the post-synaptic compartment level, individual synapses themselves exhibit functional diversity, and the cellular processes that facilitate this heterogeneity of function is currently an exciting topic of research. Unlike the mechanisms that have been described to influence specific aspects of excitatory postsynaptic plasticity, mechanisms operational at GABAergic postsynaptic terminals are relatively unexplored. Recent technological developments including single-particle tracking and super-resolution imaging demonstrate that the inhibitory post-synapse is subject to dynamic activity-dependent reorganization. Therefore, understanding the cellular mechanisms that contribute to dynamics at GABAergic synapses will help to explain emergent functional heterogeneity.

## Pre-synaptic Specification of GABAergic Plasticity

Pre-synaptically, a diverse pool of inhibitory interneurons provides GABAergic input onto post-synaptic cells. These interneurons differ in their spatial innervation patterns, firing properties, and pre-synaptic release mechanisms (Pelkey et al., 2017). Interestingly, recent data suggest that GABAergic plasticity occurs differentially between synapses innervated by distinct classes of interneurons. Pre-synaptic plasticity importantly involves regulation of neurotransmitter release onto the post-synaptic cell, often *via* modification of vesicular release (McBain and Kauer, 2009). How this released GABA is sensed and transduced to the target cell then depends on post-synaptic signaling.

**Figure 1:**
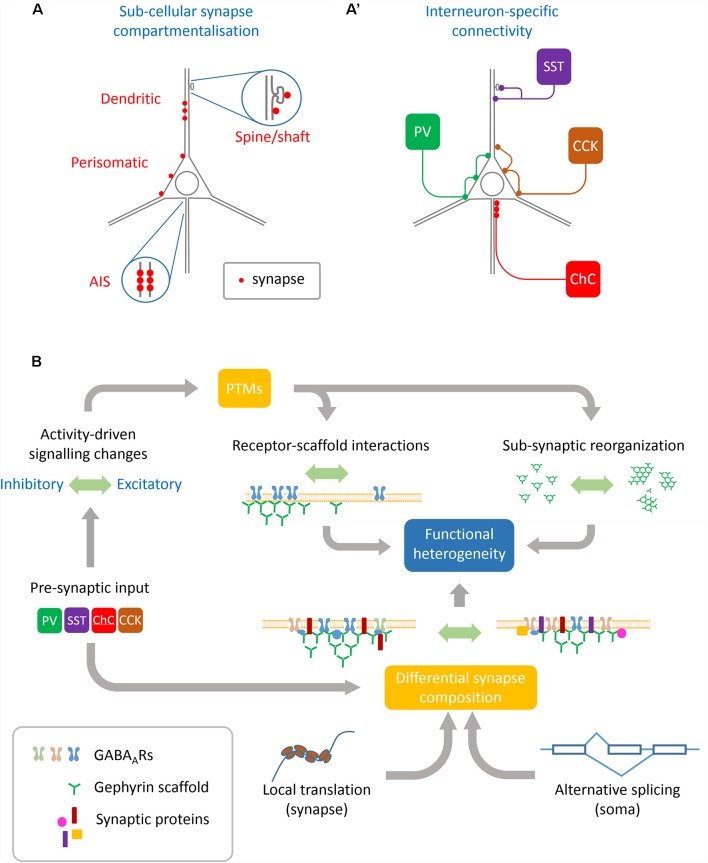
Sources of heterogeneity contributing to GABAergic synapse remodeling. **(A)** Basal synapse dynamics and responses to activity are distinct between different neuronal sub-compartments such as the axon initial segment (AIS), perisomatic and dendritic synapses, and even between inhibitory synapses situated on dendritic shafts vs. spines. **(A′)** Pre-synaptic interneuron subtypes innervate different neuronal sub-compartments. Interneuron subtypes innervating similar compartments can also differ in their functional modulation, such as between synapses innervated by PV+ or CCK+ basket cells which both target the perisomatic domain.** (B)** Many cellular mechanisms converge to achieve functional heterogeneity at GABAergic synapses: pre-synaptic interneurons specify some aspects of synaptic protein composition as well as determine pre-synaptic GABA release and plasticity. These along with other upstream signals including neuronal activation and intracellular calcium concentration can regulate post-translational modifications (PTMs) on both receptors and scaffolds which alter their dynamics as well as sub-synaptic organization. This synaptic organization is further defined by local translation of nascent proteins as well as alternate splicing of transcripts conferring specific properties to the synapse.

Distinct interneuron subclasses differentially target specific neurons and sub-cellular compartments (e.g., soma, dendritic shaft, dendritic spines, axon-initial segment, et cetera; Figures 1A,A′). For example, cholecystokinin-positive (CCK+) and parvalbumin-positive (PV+) basket cells target the soma and proximal dendrites of neurons, whereas somatostatin-positive (SST+) interneurons preferentially target both the shafts and spines of dendrites. The mechanisms specifying different innervation patterns are in part provided by the expression of specific synaptic organizers by the post-synaptic cell. At hippocampal perisomatic synapses, the dystrophin-glycoprotein complex specifically organizes inputs from CCK+ interneurons which target the peri-somatic domain (Früh et al., 2016; Panzanelli et al., 2017). This complex is absent from distal dendrites or the axon-initial segment, and genetic deletion of this complex specifically affects CCK+ terminals. In contrast, trans-synaptic organizers like L1CAM-AnkyrinG interactions specify axo-axonic synapses onto the axon initial segment (AIS), and organize the input-specific synaptic properties of chandelier cells (Tai et al., 2019). Neuroligins which mediate trans-synaptic interactions control spatial input specificity and synaptic strength depending on the neuroligin isoform expressed. While neuroligin 2 is required to form both PV+ and SST+ synapses, neuroligin 3 can selectively regulate the strength of SST+ synapses dependent on its expression level (Horn and Nicoll, 2018). Moreover, PV+ and SST+ synapses are regulated by distinct upstream signaling, with PV+ synapses being more affected by cell-autonomous firing and SST+ synapses affected by NMDA receptor (NMDAR)-driven glutamatergic input (Horn and Nicoll, 2018). In another example, activation of post-synaptic NMDARs signal downstream to the kinase CaMKIIα, which then specifically drives inhibitory long-term potentiation (iLTP) at SST+, but not PV+ synapses (Chiu et al., 2018). The subunit composition of post-synaptic GABA_A_Rs may also act as a substrate for synapse-specific plasticity between these interneuron types, as post-synaptic loss of the β3 subunit specifically affects PV+ driven input (Nguyen and Nicoll, 2018). Interneuron-specific plasticity is also represented at CCK+ synapses onto pyramidal cells, which are regulated by retrograde signaling *via* cannabinoid type-1 (CB1) receptors. These CB1 receptors are pre-synaptically enriched at CCK+ synapses and participate in the depolarization-induced suppression of inhibition (DSI; Busquets-Garcia et al., 2018). Interestingly pyramidal neuron activation was shown to affect the expression of the intermediate early gene and transcription factor NPAS4 to enhance inputs from CCK+ neurons to drive DSI but failed to enhance PV+ neuron input (Hartzell et al., 2018). This study provides a link between neuron activation status and interneuron-specific inhibition *via* transcriptional control, although which NPAS4-regulated synaptogenic targets couple activity to synapse-specific recruitment are currently undetermined. While the generality of input-specific plasticity and description of underlying mechanisms remains to be elaborated, it is clear that variation in synaptic protein composition facilitates at least some forms of pre-synaptic input specificity (Chiu et al., 2018).

## Importance of Receptor-Scaffold Interactions

The GABAergic post-synapse contains GABA_A_ receptors (GABA_A_Rs), post-synaptic scaffolding and signaling proteins, and trans-synaptic adhesion molecules which facilitate effective communication between the pre- and post-synapse for efficient neurotransmission. GABA_A_Rs are composed of pentamers from a family of subunits encoded by 19 distinct genes (subunits α1–6, β1–3, γ1–3, δ, ε, π, ρ1–3, and τ). Although it has been recently shown that many receptor subunits can access the synaptic space (Hannan et al., 2019), the select interactions between receptors and post-synaptic scaffolds such as gephyrin encourage the retention of GABA_A_Rs composed of the combination of α1–3 subunits along with β1–3 and γ2 subunits, whereas those containing the subunits α4–6 and δ tend to be extra-synaptic (Fritschy and Panzanelli, 2014; Hannan et al., 2019). GABA_A_Rs are trafficked to the plasma membrane from cytoplasmic pools, or diffuse laterally within the membrane in and out of synapses to alter the local concentration of receptors and therefore synaptic strength (Flores and Méndez, 2014; Petrini and Barberis, 2014). Thus, control over the diffusion dynamics of GABA_A_Rs is an important mechanism by which inhibitory plasticity is achieved (Petrini and Barberis, 2014). In gephyrin-containing GABAergic synapses, the magnitude of retention of GABA_A_Rs scales with the size of gephyrin clusters (Specht et al., 2013; Flores et al., 2015; Crosby et al., 2019). Consequently, knockdown of gephyrin leads to a reduction in synaptic receptors *via* decreased confinement of GABA_A_Rs (Jacob, 2005; Thomas et al., 2005). Similarly, signaling which induces gephyrin clustering is often coupled to increase in GABA_A_R clustering. For example, activity induction in hippocampal slices leads to inhibitory potentiation that is correlated to increases in gephyrin cluster size concordant with mIPSC amplitude (Flores et al., 2015). Additionally, during long-term potentiation of GABAergic synapses (iLTP), synaptic gephyrin clusters show increases in the number of gephyrin molecules at the same time that extra-synaptic clusters shrink (Pennacchietti et al., 2017). Due to the close and interrelated changes between gephyrin clustering and those of GABAergic transmission (Petrini et al., 2014; Flores et al., 2015; Specht, 2019), the analysis of changes in both gephyrin and GABA_A_R synaptic organization can be used to understand mechanistic bases for synapse alterations.

## Heterogeneity of GABAergic Post-synaptic Remodeling

While plasticity occurs at all synapses, basal synapse characteristics such as size, strength, and composition are variable, and therefore the extent of induced synaptic plasticity is also variable. For example, spinal cord synapses contain over four times as many gephyrin molecules per synapse and at a higher density than cortical synapses (Specht et al., 2013). GABAergic synaptic dynamics can even vary between compartments within the same neuron, where spine synapses are more dynamic than shaft synapses (Villa et al., 2016). Critically, the manner in which inhibitory synapses remodel depends on the valency of signaling received, where activity increases or decreases can have similar or opposing effects on synaptic dynamics. A strong pharmacological network activity increase can lead to a reduction in the clustering of gephyrin, resulting in a decrease of inhibitory synaptic currents in a NMDAR- and calcineurin-dependent manner (Bannai et al., 2009). This contrasts with data suggesting that activity increases lead to enhanced gephyrin clustering and GABA_A_R synaptic accumulation through CaMKII signaling (Flores et al., 2015). These differences have been explained by the degree of activity-induction triggering distinct calcium signaling pathways: whereas low calcium can act to stabilize gephyrin and GABA_A_Rs at synapses, large increases in calcium leads to reduced retention of GABA_A_Rs (Petrini and Barberis, 2014; Bannai et al., 2015). Moreover, after induction of activity paradigms such as iLTP, some but not all synapses show re-arrangement of their nano-domains (Pennacchietti et al., 2017), suggesting that even synaptic plasticity itself can only occur where synapse-specific mechanisms allow for it. How signaling then is organized to effect plasticity can only be understood once upstream signaling effectors or downstream signaling targets are identified.

## Multiple Signal Transduction Pathways Modulate Receptor-Scaffold Interactions

Direct modification of GABA_A_Rs, the interaction between GABA_A_Rs and post-synaptic scaffolds, or the dynamics of the post-synaptic scaffolds themselves could all contribute to modulating synaptic receptor retention and therefore the function of inhibitory synapses (Choquet and Triller, 2003; Petrini and Barberis, 2014; Specht, 2019). Post-translational modifications (PTMs) including protein phosphorylation, SUMOylation, acetylation, palmitoylation, and nitrosylation, are known to occur at the inhibitory post-synapse (Tyagarajan and Fritschy, 2014) where they can effectively function *via* altered receptor-scaffold interactions. Of these, modification of GABA_A_Rs (Comenencia-Ortiz et al., 2014; Petrini and Barberis, 2014) and gephyrin (Tyagarajan and Fritschy, 2014; Zacchi et al., 2014; Kasaragod and Schindelin, 2018) are best described. Palmitoylation of both GABA_A_Rs and gephyrin result in enhanced surface localization (Matt et al., 2019), conversely ubiquitination (Luscher et al., 2011) or SUMOylation (Ghosh et al., 2016) of these proteins results in decreased synaptic accumulation. While phosphorylation of GABA_A_Rs controls both surface trafficking and removal (Comenencia-Ortiz et al., 2014), it also influences receptor diffusion in and out of synapses *via* gephyrin-dependent (Mukherjee et al., 2011) or independent mechanisms (Lévi et al., 2015). Gephyrin itself is importantly regulated by phosphorylation, which can lead to either reduced gephyrin clustering (Tyagarajan et al., 2013), or enhanced gephyrin clustering (Flores et al., 2015) depending on the specific amino acid residue phosphorylated. Still, the molecular and biophysical mechanisms transducing these phosphorylation events to effect function are poorly understood.

Recent efforts towards describing post-synaptic dynamics have employed live-imaging and super-resolution microscopy to determine real-time and nano-scale re-organization of the post-synapse (Specht et al., 2013; Pennacchietti et al., 2017; Battaglia et al., 2018; Crosby et al., 2019). These studies demonstrate that gephyrin is arranged in nano-domains within the post-synapse, and also that it can cluster at extra-synaptic sites previously overlooked by conventional microscopy (reviewed by Specht, 2019). Recently, gephyrin nano-domains were directly shown to overlap with the nano-domains of GABA_A_Rs as well as those of pre-synaptic vesicle release sites clearly demonstrating that synaptic gephyrin nano-domains represent functional organizational units (Crosby et al., 2019). In this context, the impact of gephyrin upon GABA_A_Rs has been shown by perturbing gephyrin clustering *via* overexpression of dominant-negative gephyrin, which causes a reduction in the number and size of GABA_A_R nano-domains (Crosby et al., 2019) and functionally reduces the dwell time of GABA_A_Rs at synaptic sites (Battaglia et al., 2018).

PTMs have now been shown to control gephyrin nano-domain structure and GABA_A_R retention at synapses. A recent study has found that phosphorylation of gephyrin at serine 268 (regulated by ERK1/2; Tyagarajan et al., 2013) results in increased nano-domain compaction and a reduction in GABA_A_R synaptic dwell time (Battaglia et al., 2018). Conversely preventing phosphorylation at residue serine 270 (regulated by GSK3β or CDK5; Tyagarajan et al., 2011; Kuhse et al., 2012) causes a decrease in gephyrin scaffold compaction, while also increasing the scaffold size. Interestingly gephyrin mutations additionally altered GABA_A_R dynamics outside of synaptic sites, suggesting that gephyrin is involved in extra-synaptic receptor scaffolding regulated by phosphorylation of distinct serine residues (Battaglia et al., 2018). Taken together PTMs such as phosphorylation provide a link between upstream signaling cascades and functional plasticity at the post-synapse *via* receptor-scaffold interactions. Phospho-proteomic analyses of synaptic proteins indicate that more than just gephyrin and GABA_A_Rs are dynamically phosphorylated, and that altered brain states such as sleep deprivation (Wang et al., 2018) or induction of learning lead to broad phosphorylation changes (Kähne et al., 2016). Learning paradigms can alter the abundance of kinases and phosphatases which regulate the phospho-status of synaptic proteins including those which signal to GABA_A_Rs and gephyrin (Šmidák et al., 2016). Therefore, differential phosphorylation of inhibitory synaptic protein networks may serve as a substrate underlying synapse-specific or broader network form of plasticity.

## Synaptic Composition Changes May Drive Synapse Remodeling

Models for receptor-scaffold interactions propose that modifying the number of scaffolds or the affinity of receptor-scaffold binding will define the equilibrium governing immobilization of receptors at the synapse (Choquet and Triller, 2003; Specht, 2019). Therefore, heterogeneity in synaptic protein composition between areas of the nervous system, within microcircuits, and even within the same cell may explain resulting differences in synaptic plasticity. While the contribution of a handful of inhibitory synaptic proteins such as collybistin, gephyrin, and neuroligins to GABA_A_Rs dynamics and inhibitory synapse function have been identified (Fritschy et al., 2012; Tyagarajan and Fritschy, 2014; Groeneweg et al., 2018), recent unbiased screens have greatly expanded the pool of potential regulatory proteins. Immunoprecipitation or proximity ligation-based detection of the protein identity of post-synaptic interacting complexes has been performed for gephyrin, collybistin, InSyn1 (Uezu et al., 2016), neuroligin 2 (Kang et al., 2014), GABA receptors (Nakamura et al., 2016; Ge et al., 2018), as well as for the inhibitory synaptic cleft (Loh et al., 2016). These efforts have uncovered hundreds of novel inhibitory synaptic proteins including scaffolding proteins, kinases, and components of signal transduction cascades. For example, the tetraspanin protein LHFPL4 was identified as a novel binding partner of neuroligin 2 (Yamasaki et al., 2017), disruption of which results in severe inhibitory synapse deficits leading to death (Wu et al., 2018). Interestingly this protein was shown to mediate cell-types-specific regulation, affecting synapses in pyramidal cells but not interneurons (Davenport et al., 2017). Comparative analysis of proteomes between excitatory synapses have shown regional (Roy et al., 2018), activity-, and state-dependent alterations in plasticity proteins (Lautz et al., 2018). Currently, similar condition-dependent information specific to GABAergic synapses is lacking, and moreover how the protein composition of these synapse is modified dynamically is only starting to be understood.

## Post-transcriptional Control Over GABAergic Synapses

Recent data suggests that local translation of mRNA coding for synaptic proteins could offer a way to acutely modify synaptic composition in a synapse-specific manner (Rangaraju et al., 2017). In fact, a plethora of inhibitory synaptic mRNA transcripts have been identified as present at the synapse including those coding for GABA_A_Rs and adaptor proteins (Cajigas et al., 2012; Zappulo et al., 2017). Recently, it was found that 75% of inhibitory synaptic terminals possess translational machinery, and 40% of these terminals exhibit active translation at a given time (Hafner et al., 2019), although the identity and inhibitory synapse specificity of these newly-translated proteins are unknown. Functionally, disruption of the localization of synaptic mRNA transcripts can affect synapse organization. For example, synaptic accumulation of mRNA coding for the α2 GABA_A_R subunit is disrupted in a loss-of-function mouse model null for the RNA binding protein NONO, leading to a reduction in synaptic GABA_A_Rs and gephyrin clustering (Mircsof et al., 2015). Alternative splicing of mRNA coding for synaptic proteins provides an additional mechanism to generate heterogeneity in synaptic signaling. Splicing of neurexins has been shown to be important for excitatory synapse specification, differentially affecting NMDAR or AMPAR driven transmission (Dai et al., 2019), and leading to synaptic and behavioral dysfunction when splicing is disrupted (Traunmüller et al., 2016). Recently, alternative splicing of inhibitory synaptic proteins was shown to coordinate spatial GABAergic synapse organization. Splice isoforms of collybistin, a core component of inhibitory synapses was found to control dendritic inhibitory synapse patterning along the proximal-distal axis (de Groot et al., 2017). Collybistin was later identified as a target for alternative splicing by the RNA binding protein Sam68, which was also shown to control splicing of gephyrin mRNA at the C4 splice cassette known to control post-synaptic clustering (Witte et al., 2019). Whether splicing of mRNA coding for inhibitory proteins occurs locally at individual synaptic sites and contributes to synapse-specific protein composition is currently unknown.

## Conclusion

The findings highlighted in this mini-review article (summarized in Figure 1B) reveals a shift in thinking about how inhibitory synaptic plasticity occurs. Beyond simple measurements of changes in post-synaptic currents, advances in microscopic imaging technology, RNA sequencing, mass spectrometry, and molecular visualization tools enable the investigation of how plasticity manifests within and between individual synapses. While future interrogation of plasticity will undoubtedly uncover new mechanisms underlying synapse remodeling, they also allow us to fully appreciate the heterogeneity in synaptic function, between different brain circuits, neuronal compartments, individual synapses, and now even within sub-synaptic nano-domains.

## Author Contributions

BC and ST contributed to the organization and writing of this manuscript.

## Conflict of Interest Statement

The authors declare that the research was conducted in the absence of any commercial or financial relationships that could be construed as a potential conflict of interest.
